# Editorial: Exploring Reliable Markers and Prediction Indexes for the Progression From Subjective Cognitive Decline to Cognitive Impairment

**DOI:** 10.3389/fnagi.2021.760920

**Published:** 2021-09-20

**Authors:** Jiehui Jiang, Ying Han, Frank Jessen

**Affiliations:** ^1^Institute of Biomedical Engineering, School of Information and Communication Engineering, Shanghai University, Shanghai, China; ^2^Department of Neurology, Xuanwu Hospital of Capital Medical University, Beijing, China; ^3^Department of Psychiatry, University of Cologne, Cologne, Germany

**Keywords:** preclinical Alzheimer's disease, subjective cognitive decline, marker, neuroimaging, prediction indexes

Alzheimer's disease (AD) is a neurodegenerative disorder and the most common cause of dementia. There is currently no effective treatment, which makes preclinical prediction for AD particularly important (Huang et al., 2020). Subjective cognitive decline (SCD) has been proposed as important preclinical stages in the development of AD (Sperling et al., 2011). A growing body of evidence shows that SCD may be one of the earliest noticeable symptoms of AD and related dementias. Therefore, it is required to explore reliable biomarkers and prediction indexes for patients with high progression risks from SCD to cognitive impairment.

Taking this into consideration, the Research Topic “Exploring Reliable Markers and Prediction Indexes for the Progression from Subjective Cognitive Decline to Cognitive Impairment” by Frontiers in Aging Neuroscience makes a contribution with updates and different perspectives on this important theme, developed over 19 papers. These updates focus on exploring reliable markers and prediction indexes for the progression of SCD from multidisciplinary perspectives including neuroimaging techniques, genetic or inflammation mechanisms, as well as Artificial Intelligence (AI) applications.

The author Wang X. et al., focus on subjects with low and high plasma Aβ levels among individual with SCD. They investigate the microstructural changes in white matter (WM) based on diffusion tensor imaging from dataset of Sino Longitudinal Study on Cognitive Decline (SILCODE). Result shows a correlation between WM integrity (e.g., fractional anisotropy and mean diffusivity) and plasma β-amyloid (Aβ) 40 levels rather than Aβ42 in individuals with SCD. This indicates plasma Aβ40 levels may represent a useful biomarker to predict different trajectories of aging in individuals with SCD.

Another case-control study by Qiao et al., analyses the associations between WM disruptions and cognitive declines at the early stage of subcortical vascular cognitive impairment (SVCI). This study concludes the damage of long WM in right hemisphere in the pre-SVCI patients and correlated with declines in executive functions and spatial processing.

The study by Huang et al., uses multi-kernel support vector machine (SVM) to examine whether WM structural networks can be used for screening SCD and aMCI. Their findings promote the development of potential brain imaging markers for the early detection of AD.

Based on diffusional kurtosis imaging (DKI) and three-dimensional (3D) arterial spin labeling (ASL), Yang et al., explore microstructural and cerebral blood flow (CBF) abnormalities in individuals with SCD plus and aMCI. They point out the mean kurtosis of DKI may be used as an early potential neuroimaging biomarker and may be more sensitive than CBF at the very early stage of AD.

The paper by Wu Z. et al., examines group differences in gray matter surface morphometry, including cortical thickness, the gyrification index (GI), and the sulcus depth. The authors aim to track the progression of the disease in different stages of AD, including health controls, early MCIs, late MCIs, and ADs. Based on region-of-interest (ROI) analysis, their study shows that cortical thickness and sulcus depth indices are predominant during AD progression while GI is insensitive. The findings highlight the relevance between gray matter surface morphometry and the stages of AD, laying the foundation for *in vivo* tracking of AD progression.

The study by Fu et al., extracts gray matter volumes to predict the regional densities in the whole brain in normal control (NC), SCD, Amnestic mild cognitive impairment (aMCI) and AD. In this study, decreased structural covariance and weakened connectivity strength are observed in SCD compared with NC. In addition, increased structural covariance in aMCI and decreased structural covariance in AD are also found. These results provide evidence to the structural disconnection hypothesis in individuals with SCD.

The study by Li et al., points out the impairment in spatial navigation (SN) in patients with MCI. They demonstrate that structural connectivity network abnormalities, especially in the frontal and parietal gyri, are associated with a lower SN accuracy, independently of white matter hyper intensities, which providing a new insight into the brain mechanisms associated with SN impairment in MCI.

The study by Cui et al., points out different functional activity of the SCD patients with aMCI patients, which suggest SCD may be a separate stage of cognitive decline before aMCI and is helpful to the study of preclinical cognitive decline.

Based on the topological characteristics of the WM network, Tao et al., further identify individuals with SCD or aMCI from healthy control (HC) and to describe the relationship of pathological changes in these two stages. They conclude that the neural degeneration from SCD to aMCI follows a gradual process, from abnormalities at the nodal level to those at both nodal and network levels.

The study by Chen Q. et al., identifies distinct functional states and explore the reconfiguration functional connectivity (FC) in individuals with SCD. Results indicate that the alterations of dynamic FC may underlie the early cognitive decline in SCD patients and could be served as sensitive neuroimaging biomarkers.

Taking the important role of self-reference processing into account, Wei et al., discover four interactions among self-reference network (SRN), dorsal attention network (DAN), and salience network (SN) using resting-state fMRI. These results point out that the influence of the SRN in the ultra-early stages of AD is non-negligible.

The study by Xu et al., explores the specific characteristic based on the multimodal brain networks, including individual morphological, structural and functional brain networks. Results highlight the role of cortical-subcortical circuit in individuals with SCD, providing potential biomarkers for the diagnosis and prediction of the preclinical stage of AD.

The study by Wu L. et al., investigates the cognitive impairment in individuals with chronic pontine stroke based on voxel-mirrored homotopic connectivity. Results indicate the important role of lingual gyrus and precuneus as ROIs in the early diagnosis of cognitive impairment individuals with chronic pontine stroke.

The study by Wang Y. et al., demonstrates that the carotid calcifications are associated with post-stroke cognitive impairment (PSCI). They conclude that the significant role of large vessel atherosclerosis in PSCI should be concerned in future study.

The study by Chen Y. et al., concludes that the methylation of peripheral NCAPH2 could be used as a useful peripheral biomarker in the early stage of AD screening. Low levels of NCAPH2 methylation are observed in SCD, and which is independent of the APOE ε4 status. In addition, there is a positive correlation between NCAPH2 methylation levels and the hippocampal volumes in SCD APOE ε4 non-carriers.

The study by Dakterzada et al., compares the results of Innotest enzyme-linked immunoassay (ELISA) with two automated methods (Lumipulse and Elecsys). Both Lumipulse and Elecsys methods are highly concordant with clinical diagnoses, and the combination of Lumipulse Ab42 and P-tau has the highest discriminating power. They recommend both automated methods for the measurement of CSF biomarkers.

The study by Shi et al., explores whether adenosine receptor 1 (A1 R) is involved in electroacupuncture (EA) pretreatment induced cognitive impairment after focal cerebral ischemia in rats. The results showed that EA pretreatment revered cognitive impairment, improved neurological outcome, and inhibited apoptosis at 24 h after reperfusion. Pretreatment with CCPA (a selective A1 receptor agonist) could imitate the beneficial effects.

The study by Lin et al., examines the relationship between spinal cord injury (SCI) and olfactory dysfunction. They point out that the SCI initiates pathological processes, including inflammatory response and impaired neurogenesis. These results provide a basis for pathological mechanisms of early neurodegenerative diseases involving the olfactory bulb and enable early clinical drug intervention.

Essential tremor (ET) is occasionally associated with a high risk for MCI and dementia. The retrospective study by Wu P. et al., proposes the sustained clinical efficacy of unilateral magnetic resonance-guided focused ultrasound (MRgFUS) thalamotomy in Chinese patients with ET.

## Author Contributions

JJ, YH, and FJ have written this editorial for the Research Topic they have edited. All authors contributed to the article and approved the submitted version.

## Conflict of Interest

The authors declare that the research was conducted in the absence of any commercial or financial relationships that could be construed as a potential conflict of interest.

## Publisher's Note

All claims expressed in this article are solely those of the authors and do not necessarily represent those of their affiliated organizations, or those of the publisher, the editors and the reviewers. Any product that may be evaluated in this article, or claim that may be made by its manufacturer, is not guaranteed or endorsed by the publisher.
